# (2*E*)-1-(2,4-Dimethyl­quinolin-3-yl)-3-phenyl­prop-2-en-1-one

**DOI:** 10.1107/S1600536813004765

**Published:** 2013-02-23

**Authors:** R. Prasath, P. Bhavana, Seik Weng Ng, Edward R. T. Tiekink

**Affiliations:** a Department of Chemistry, BITS, Pilani–K. K. Birla Goa Campus, Goa 403 726, India; b Department of Chemistry, University of Malaya, 50603 Kuala Lumpur, Malaysia; c Chemistry Department, Faculty of Science, King Abdulaziz University, PO Box 80203 Jeddah, Saudi Arabia

## Abstract

Two independent mol­ecules comprise the asymmetric unit of the title compound, C_20_H_17_NO, which differ in the orientation of the terminal phenyl ring with respect to the quinoline ring [the dihedral angles are 75.72 (11) and 84.53 (12)° for the two mol­ecules]. The conformation about each of the ethyl­ene bonds [1.329 (3) and 1.318 (3) Å] is *E*. The crystal structure features a combination of C—H⋯N, C—H⋯π and π–π contacts [inter-centroid between the phenyl ring and the quinoline benzene ring is 3.6024 (19) Å], generating a three-dimensional network.

## Related literature

For background details and the biological application of quinoline and quinoline chalcones, see: Joshi *et al.* (2011 ▶); Prasath & Bhavana (2012 ▶); Kalanithi *et al.* (2012 ▶); Prasath *et al.* (2013 ▶). For the structures of the isomorphous chloro- and methyl-benzene derivatives, see: see: Prasath *et al.* (2011 ▶, 2012 ▶).
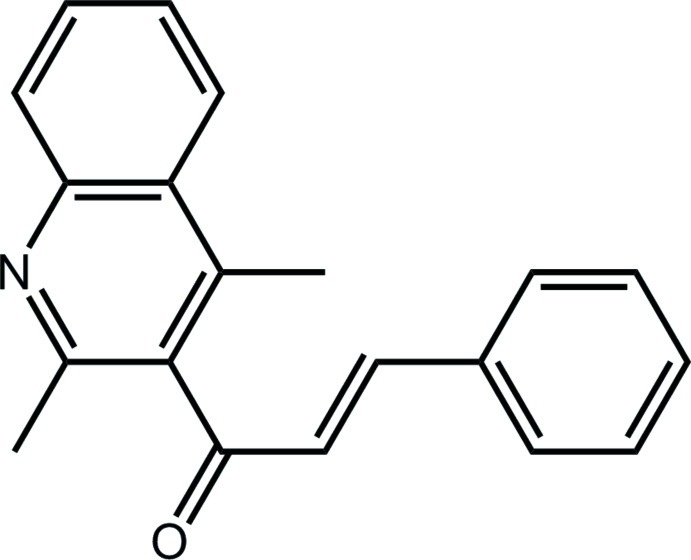


## Experimental

### 

#### Crystal data

C_20_H_17_NO
*M* *_r_* = 287.35
Triclinic, 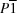

*a* = 11.1295 (9) Å
*b* = 11.5764 (8) Å
*c* = 13.3989 (11) Å
α = 96.176 (6)°
β = 112.900 (8)°
γ = 96.533 (6)°
*V* = 1558.0 (2) Å^3^
*Z* = 4
Mo *K*α radiation
μ = 0.08 mm^−1^
*T* = 295 K
0.30 × 0.20 × 0.10 mm

#### Data collection

Agilent SuperNova Dual diffractometer with an Atlas detector
Absorption correction: multi-scan (*CrysAlis PRO*; Agilent, 2011 ▶) *T*_min_ = 0.764, *T*_max_ = 1.000
14138 measured reflections
7191 independent reflections
3395 reflections with *I* > 2σ(*I*)
*R*_int_ = 0.031

#### Refinement

*R*[*F* ^2^ > 2σ(*F* ^2^)] = 0.066
*wR*(*F* ^2^) = 0.190
*S* = 1.05
7191 reflections
401 parameters
H-atom parameters constrained
Δρ_max_ = 0.14 e Å^−3^
Δρ_min_ = −0.16 e Å^−3^

### 

Data collection: *CrysAlis PRO* (Agilent, 2011 ▶); cell refinement: *CrysAlis PRO*; data reduction: *CrysAlis PRO*; program(s) used to solve structure: *SHELXS97* (Sheldrick, 2008 ▶); program(s) used to refine structure: *SHELXL97* (Sheldrick, 2008 ▶); molecular graphics: *ORTEP-3 for Windows* (Farrugia, 2012 ▶), *QMol* (Gans & Shalloway, 2001 ▶) and *DIAMOND* (Brandenburg, 2006 ▶); software used to prepare material for publication: *publCIF* (Westrip, 2010 ▶).

## Supplementary Material

Crystal structure: contains datablock(s) global, I. DOI: 10.1107/S1600536813004765/hg5294sup1.cif

Structure factors: contains datablock(s) I. DOI: 10.1107/S1600536813004765/hg5294Isup2.hkl

Supplementary material file. DOI: 10.1107/S1600536813004765/hg5294Isup3.cml

Additional supplementary materials:  crystallographic information; 3D view; checkCIF report

## Figures and Tables

**Table 1:** Hydrogen-bond geometry (Å, °) *Cg*1 and *Cg*2 are the centroids of the C1–C6 and C15–C20 rings, respectively.

*D*—H⋯*A*	*D*—H	H⋯*A*	*D*⋯*A*	*D*—H⋯*A*
C14—H14⋯N2	0.93	2.59	3.463 (3)	156
C7—H7*C*⋯*Cg*1^i^	0.96	2.86	3.662 (3)	142
C39—H39⋯*Cg*2^ii^	0.93	2.88	3.679 (3)	145

Symmetry codes: (i) 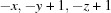; (ii) 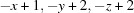.

**Fig. 1.**
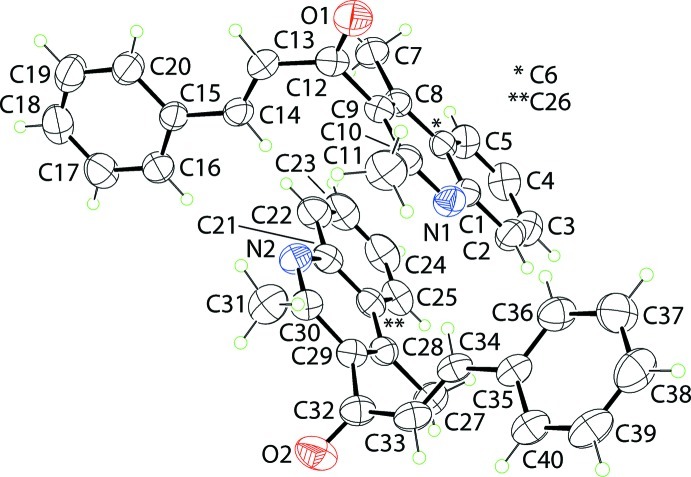
The molecular structure of the two independent molecules in (I) showing the atom-labelling scheme and displacement ellipsoids at the 35% probability level.

**Fig. 2.**
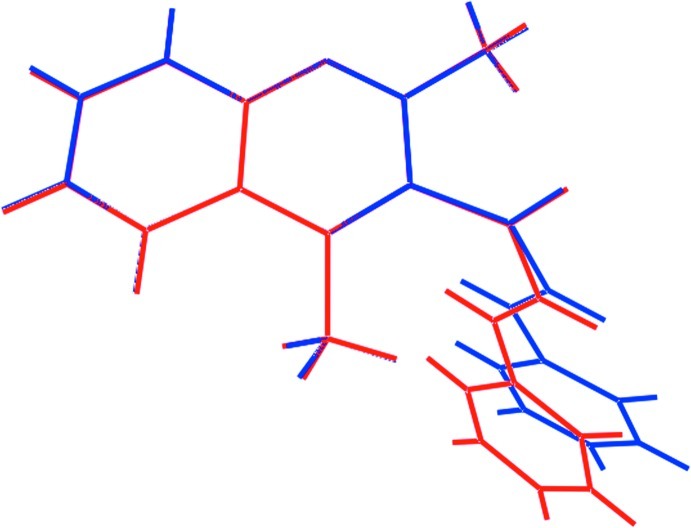
Overlay diagram of the two independent molecules in (I) with the N1-containing molecule illustrated in red. The molecules are overlaid so that the pyridyl rings are superimposed.

**Fig. 3.**
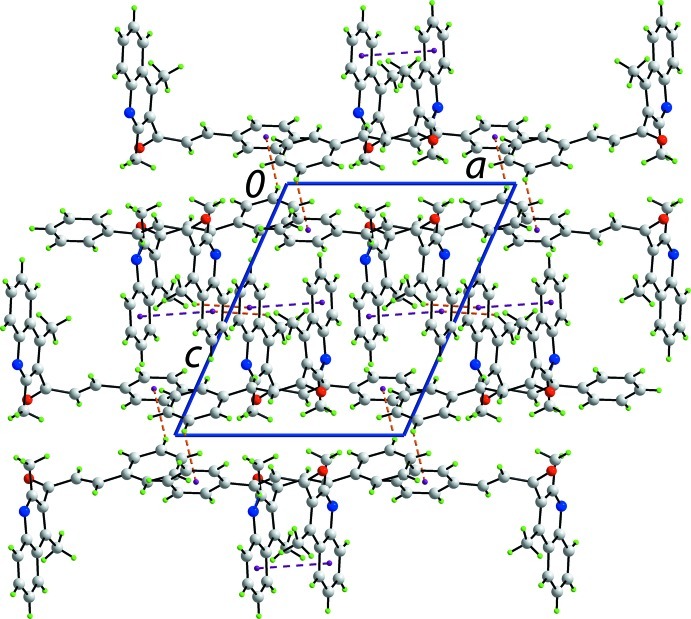
A view in projection down the b axis of the unit-cell contents of (I). The C—H···N, C—H···O, C—H···π, and π···π interactions are shown as blue, orange and purple dashed lines, respectively.

## supplementary crystallographic information

### Comment

Quinoline and their corresponding heterocyclic analogues are valuable intermediates in organic synthesis and exhibit a multitude of biological activities. (Prasath & Bhavana, 2012; Joshi *et al.*, 2011). Quinoline chalcone analogues have also gained much attention due to their bioactivity such as anti-plasmodial, anti-microbial, anti-malarial and anti-cancer activities (Prasath *et al.*, 2013; Kalanithi *et al.*, 2012). In was in this connection, that the title compound, (I), was investigated.

Two crystallographically independent molecules comprise the asymmetric unit of (I), Fig. 1. As clearly indicated in Fig. 2, an overlay diagram of the molecules, the major difference between them is manifested in the dihedral angles formed by the ten atoms of the quinolinyl ring (r.m.s. deviations = 0.010 and 0.013 Å) and the terminal phenyl ring, *i.e*. 75.72 (11)° for the N1-containing molecule and 84.53 (12)° for the N2-containing molecule. The overall conformation of each molecule is therefore of the letter *L*. The configuration around each ethylene bond [C13═C14 = 1.329 (3) Å and C33═C34 = 1.318 (3) Å] is *E*. The overall molecular conformation found for the molecules comprising (I) match those of the chloro- (Prasath *et al.*, 2011) and methyl-benzene (Prasath *et al.*, 2012) analogues; the three structures are in fact isomorphous.

The three-dimensional architecture is stabilized by a combination of ethylene-C—H···N2 interactions [between the independent molecules] and C—H···π interactions between methyl-H and a C_6_ ring of the N1-quinolinyl residue, and between phenyl-H and the phenyl (C15–C20) ring, Table 1, along with π···π contacts between the independent molecules [inter-centroid distance = 3.6024 (19) Å, angle of inclination = 2.43 (15)°], *i.e*. between the C_6_ ring of the N1-quinolinyl residue and the phenyl (C35–C40) ring of the N2-containing molecule, Fig. 3.

### Experimental

A mixture of 3-acetyl-2,4-dimethylquinoline (1.0 g, 0.005 *M*), benzaldehyde (530 mg, 0.005 *M*) and KOH (0.5 g) in distilled ethanol (50 ml) was stirred for 12 h at room temperature. The resulting mixture was neutralized with dilute acetic acid. The resultant solid was filtered, dried and purified by column chromatography using a 1:1 mixture of ethyl acetate and hexane. Re-crystallization was by slow evaporation of an acetone solution of (I) which yielded colourless prisms in 80% yield; *M*.pt: 421–423 K.

### Refinement

The C-bound H atoms were geometrically placed (C—H = 0.95–0.96 Å) and refined as riding with *U_iso_*(H) = 1.2–1.5*U_eq_*(C). Owing to poor agreement, one reflection, *i.e*. (0 0 10), was removed from the final cycles of refinement.

### Crystal data

**Table:**

C_20_H_17_NO	*Z* = 4
*M**_r_* = 287.35	*F*(000) = 608
Triclinic, *P*1	*D*_x_ = 1.225 Mg m^−^^3^
Hall symbol: -P 1	Mo *K*α radiation, λ = 0.71073 Å
*a* = 11.1295 (9) Å	Cell parameters from 2234 reflections
*b* = 11.5764 (8) Å	θ = 2.9–27.5°
*c* = 13.3989 (11) Å	µ = 0.08 mm^−^^1^
α = 96.176 (6)°	*T* = 295 K
β = 112.900 (8)°	Prism, colourless
γ = 96.533 (6)°	0.30 × 0.20 × 0.10 mm
*V* = 1558.0 (2) Å^3^	

### Data collection

**Table:**

Agilent SuperNova Dual diffractometer with an Atlas detector	7191 independent reflections
Radiation source: SuperNova (Mo) X-ray Source	3395 reflections with *I* > 2σ(*I*)
Mirror monochromator	*R*_int_ = 0.031
Detector resolution: 10.4041 pixels mm^-1^	θ_max_ = 27.6°, θ_min_ = 2.9°
ω scan	*h* = −14→12
Absorption correction: multi-scan (*CrysAlis PRO*; Agilent, 2011)	*k* = −15→15
*T*_min_ = 0.764, *T*_max_ = 1.000	*l* = −17→17
14138 measured reflections	

### Refinement

**Table:**

Refinement on *F*^2^	Primary atom site location: structure-invariant direct methods
Least-squares matrix: full	Secondary atom site location: difference Fourier map
*R*[*F*^2^ > 2σ(*F*^2^)] = 0.066	Hydrogen site location: inferred from neighbouring sites
*wR*(*F*^2^) = 0.190	H-atom parameters constrained
*S* = 1.05	*w* = 1/[σ^2^(*F*_o_^2^) + (0.0564*P*)^2^ + 0.2605*P*] where *P* = (*F*_o_^2^ + 2*F*_c_^2^)/3
7191 reflections	(Δ/σ)_max_ = 0.001
401 parameters	Δρ_max_ = 0.14 e Å^−^^3^
0 restraints	Δρ_min_ = −0.16 e Å^−^^3^

### Special details

**Table:**

Geometry. All e.s.d.'s (except the e.s.d. in the dihedral angle between two l.s. planes) are estimated using the full covariance matrix. The cell e.s.d.'s are taken into account individually in the estimation of e.s.d.'s in distances, angles and torsion angles; correlations between e.s.d.'s in cell parameters are only used when they are defined by crystal symmetry. An approximate (isotropic) treatment of cell e.s.d.'s is used for estimating e.s.d.'s involving l.s. planes.
Refinement. Refinement of *F*^2^ against ALL reflections. The weighted *R*-factor *wR* and goodness of fit *S* are based on *F*^2^, conventional *R*-factors *R* are based on *F*, with *F* set to zero for negative *F*^2^. The threshold expression of *F*^2^ > σ(*F*^2^) is used only for calculating *R*-factors(gt) *etc*. and is not relevant to the choice of reflections for refinement. *R*-factors based on *F*^2^ are statistically about twice as large as those based on *F*, and *R*- factors based on ALL data will be even larger.

### Fractional atomic coordinates and isotropic or equivalent isotropic displacement parameters (Å^2^)

**Table:**

	*x*	*y*	*z*	*U*_iso_*/*U*_eq_	
O1	0.2858 (2)	0.45826 (19)	0.85864 (17)	0.0966 (7)	
O2	0.5540 (2)	1.20890 (18)	0.82791 (16)	0.0943 (7)	
N1	0.1744 (2)	0.77004 (18)	0.71898 (16)	0.0621 (5)	
N2	0.5102 (2)	0.83929 (19)	0.69801 (17)	0.0668 (6)	
C1	0.1282 (2)	0.7394 (2)	0.60732 (19)	0.0551 (6)	
C2	0.0550 (3)	0.8160 (2)	0.5418 (2)	0.0696 (7)	
H2	0.0401	0.8844	0.5752	0.084*	
C3	0.0063 (3)	0.7909 (3)	0.4306 (2)	0.0789 (8)	
H3	−0.0406	0.8426	0.3884	0.095*	
C4	0.0262 (3)	0.6876 (3)	0.3792 (2)	0.0818 (9)	
H4	−0.0089	0.6703	0.3029	0.098*	
C5	0.0965 (3)	0.6123 (3)	0.4400 (2)	0.0710 (7)	
H5	0.1095	0.5442	0.4048	0.085*	
C6	0.1501 (2)	0.6361 (2)	0.55593 (18)	0.0543 (6)	
C7	0.2529 (3)	0.4502 (2)	0.5729 (2)	0.0774 (8)	
H7A	0.2966	0.4060	0.6292	0.116*	
H7B	0.3085	0.4704	0.5355	0.116*	
H7C	0.1708	0.4034	0.5213	0.116*	
C8	0.2260 (2)	0.5615 (2)	0.62422 (19)	0.0555 (6)	
C9	0.2710 (2)	0.5937 (2)	0.73606 (19)	0.0567 (6)	
C10	0.2425 (2)	0.6994 (2)	0.78072 (19)	0.0607 (6)	
C11	0.2909 (3)	0.7357 (3)	0.9032 (2)	0.0895 (10)	
H11A	0.2705	0.8123	0.9186	0.134*	
H11B	0.3849	0.7385	0.9372	0.134*	
H11C	0.2480	0.6796	0.9318	0.134*	
C12	0.3442 (3)	0.5159 (2)	0.8141 (2)	0.0686 (7)	
C13	0.4819 (3)	0.5084 (2)	0.8351 (2)	0.0721 (8)	
H13	0.5203	0.4516	0.8752	0.086*	
C14	0.5556 (2)	0.5778 (2)	0.80014 (18)	0.0606 (6)	
H14	0.5168	0.6371	0.7644	0.073*	
C15	0.6909 (2)	0.5715 (2)	0.81151 (18)	0.0591 (6)	
C16	0.7526 (3)	0.6520 (2)	0.7698 (2)	0.0729 (7)	
H16	0.7095	0.7120	0.7392	0.088*	
C17	0.8772 (3)	0.6449 (3)	0.7728 (2)	0.0861 (9)	
H17	0.9166	0.6993	0.7437	0.103*	
C18	0.9427 (3)	0.5574 (3)	0.8188 (3)	0.0898 (10)	
H18	1.0267	0.5525	0.8213	0.108*	
C19	0.8834 (3)	0.4780 (3)	0.8606 (2)	0.0908 (10)	
H19	0.9275	0.4187	0.8916	0.109*	
C20	0.7590 (3)	0.4839 (3)	0.8577 (2)	0.0779 (8)	
H20	0.7205	0.4288	0.8869	0.093*	
C21	0.4459 (2)	0.8296 (2)	0.5867 (2)	0.0631 (7)	
C22	0.4524 (3)	0.7285 (3)	0.5211 (2)	0.0821 (9)	
H22	0.4984	0.6713	0.5546	0.099*	
C23	0.3929 (3)	0.7139 (3)	0.4106 (3)	0.0962 (11)	
H23	0.3986	0.6470	0.3686	0.115*	
C24	0.3232 (3)	0.7981 (4)	0.3590 (2)	0.0924 (11)	
H24	0.2823	0.7872	0.2827	0.111*	
C25	0.3142 (3)	0.8969 (3)	0.4195 (2)	0.0792 (9)	
H25	0.2675	0.9527	0.3838	0.095*	
C26	0.3752 (2)	0.9158 (2)	0.53583 (18)	0.0595 (7)	
C27	0.2962 (3)	1.1107 (3)	0.5540 (2)	0.0836 (9)	
H27A	0.3050	1.1732	0.6109	0.125*	
H27B	0.2044	1.0772	0.5136	0.125*	
H27C	0.3312	1.1415	0.5053	0.125*	
C28	0.3719 (2)	1.0167 (2)	0.60481 (19)	0.0591 (6)	
C29	0.4386 (2)	1.0258 (2)	0.71629 (18)	0.0569 (6)	
C30	0.5075 (3)	0.9341 (2)	0.76044 (19)	0.0629 (7)	
C31	0.5793 (3)	0.9411 (3)	0.8827 (2)	0.0888 (9)	
H31A	0.6148	0.8701	0.8980	0.133*	
H31B	0.5188	0.9504	0.9169	0.133*	
H31C	0.6502	1.0074	0.9113	0.133*	
C32	0.4519 (3)	1.1361 (2)	0.7940 (2)	0.0674 (7)	
C33	0.3464 (3)	1.1558 (2)	0.8286 (2)	0.0712 (7)	
H33	0.3522	1.2301	0.8660	0.085*	
C34	0.2431 (3)	1.0756 (2)	0.81075 (18)	0.0612 (6)	
H34	0.2377	1.0022	0.7720	0.073*	
C35	0.1350 (3)	1.0909 (2)	0.84622 (18)	0.0606 (6)	
C36	0.0303 (3)	0.9987 (3)	0.8156 (2)	0.0750 (8)	
H36	0.0306	0.9286	0.7749	0.090*	
C37	−0.0747 (3)	1.0094 (3)	0.8446 (3)	0.0931 (10)	
H37	−0.1458	0.9477	0.8215	0.112*	
C38	−0.0734 (4)	1.1121 (3)	0.9081 (3)	0.0946 (10)	
H38	−0.1433	1.1195	0.9286	0.114*	
C39	0.0312 (4)	1.2033 (3)	0.9408 (2)	0.0876 (9)	
H39	0.0323	1.2722	0.9840	0.105*	
C40	0.1341 (3)	1.1932 (2)	0.9103 (2)	0.0736 (8)	
H40	0.2042	1.2557	0.9327	0.088*	

### Atomic displacement parameters (Å^2^)

**Table:**

	*U*^11^	*U*^22^	*U*^33^	*U*^12^	*U*^13^	*U*^23^
O1	0.0916 (14)	0.0927 (16)	0.1211 (16)	0.0181 (12)	0.0506 (13)	0.0510 (13)
O2	0.1028 (15)	0.0754 (14)	0.1009 (14)	−0.0096 (12)	0.0510 (12)	−0.0128 (11)
N1	0.0681 (13)	0.0553 (13)	0.0597 (12)	0.0130 (10)	0.0248 (10)	−0.0013 (10)
N2	0.0763 (14)	0.0619 (14)	0.0660 (13)	0.0166 (11)	0.0325 (11)	0.0076 (10)
C1	0.0521 (13)	0.0514 (14)	0.0623 (14)	0.0077 (11)	0.0249 (12)	0.0058 (11)
C2	0.0700 (17)	0.0669 (18)	0.0777 (18)	0.0212 (14)	0.0328 (14)	0.0148 (14)
C3	0.0736 (18)	0.093 (2)	0.0800 (19)	0.0274 (16)	0.0336 (16)	0.0295 (17)
C4	0.0781 (19)	0.109 (3)	0.0582 (15)	0.0156 (18)	0.0277 (15)	0.0150 (17)
C5	0.0753 (18)	0.0787 (19)	0.0593 (15)	0.0157 (15)	0.0303 (14)	−0.0017 (14)
C6	0.0508 (13)	0.0525 (15)	0.0597 (14)	0.0049 (11)	0.0259 (11)	0.0005 (11)
C7	0.0805 (18)	0.0576 (17)	0.0913 (19)	0.0148 (14)	0.0355 (15)	−0.0045 (14)
C8	0.0510 (13)	0.0488 (14)	0.0660 (15)	0.0046 (11)	0.0272 (12)	−0.0023 (11)
C9	0.0545 (14)	0.0482 (14)	0.0642 (14)	0.0032 (11)	0.0228 (12)	0.0070 (11)
C10	0.0610 (15)	0.0572 (16)	0.0604 (14)	0.0056 (12)	0.0246 (12)	0.0001 (12)
C11	0.102 (2)	0.088 (2)	0.0618 (16)	0.0105 (18)	0.0234 (16)	−0.0080 (14)
C12	0.0706 (18)	0.0555 (17)	0.0766 (17)	0.0064 (13)	0.0274 (14)	0.0124 (13)
C13	0.0694 (17)	0.0611 (17)	0.0841 (18)	0.0190 (14)	0.0235 (14)	0.0271 (14)
C14	0.0642 (16)	0.0484 (15)	0.0606 (14)	0.0123 (12)	0.0167 (12)	0.0039 (11)
C15	0.0572 (15)	0.0506 (15)	0.0583 (14)	0.0127 (12)	0.0134 (12)	−0.0021 (11)
C16	0.0697 (18)	0.0649 (18)	0.0825 (18)	0.0135 (14)	0.0307 (15)	0.0043 (14)
C17	0.077 (2)	0.084 (2)	0.101 (2)	0.0089 (18)	0.0449 (17)	−0.0033 (17)
C18	0.0663 (19)	0.093 (3)	0.091 (2)	0.0177 (19)	0.0217 (17)	−0.0255 (18)
C19	0.078 (2)	0.084 (2)	0.091 (2)	0.0321 (18)	0.0123 (18)	−0.0010 (18)
C20	0.0750 (19)	0.0688 (19)	0.0775 (17)	0.0197 (15)	0.0167 (15)	0.0082 (14)
C21	0.0641 (16)	0.0623 (17)	0.0658 (16)	0.0015 (13)	0.0346 (13)	0.0000 (13)
C22	0.086 (2)	0.073 (2)	0.087 (2)	0.0064 (16)	0.0429 (17)	−0.0119 (15)
C23	0.091 (2)	0.100 (3)	0.090 (2)	−0.003 (2)	0.045 (2)	−0.030 (2)
C24	0.086 (2)	0.118 (3)	0.0609 (17)	−0.009 (2)	0.0319 (16)	−0.0170 (19)
C25	0.0693 (18)	0.100 (2)	0.0631 (16)	−0.0008 (16)	0.0279 (14)	0.0053 (16)
C26	0.0583 (15)	0.0656 (17)	0.0552 (14)	−0.0017 (13)	0.0287 (12)	0.0037 (12)
C27	0.092 (2)	0.081 (2)	0.0828 (18)	0.0267 (17)	0.0344 (16)	0.0262 (16)
C28	0.0580 (14)	0.0590 (16)	0.0655 (15)	0.0072 (12)	0.0309 (12)	0.0124 (12)
C29	0.0636 (15)	0.0546 (15)	0.0568 (14)	0.0074 (12)	0.0303 (12)	0.0070 (11)
C30	0.0706 (16)	0.0618 (17)	0.0594 (14)	0.0116 (13)	0.0302 (13)	0.0082 (12)
C31	0.105 (2)	0.096 (2)	0.0651 (17)	0.0278 (18)	0.0304 (16)	0.0164 (15)
C32	0.0830 (19)	0.0581 (17)	0.0673 (15)	0.0107 (15)	0.0378 (15)	0.0082 (13)
C33	0.091 (2)	0.0516 (16)	0.0747 (16)	0.0166 (15)	0.0388 (15)	0.0007 (12)
C34	0.0803 (17)	0.0479 (14)	0.0583 (14)	0.0184 (13)	0.0299 (13)	0.0065 (11)
C35	0.0791 (17)	0.0566 (16)	0.0535 (13)	0.0240 (14)	0.0305 (13)	0.0133 (11)
C36	0.094 (2)	0.0649 (18)	0.0807 (18)	0.0198 (16)	0.0505 (16)	0.0073 (14)
C37	0.099 (2)	0.088 (2)	0.112 (2)	0.0121 (19)	0.066 (2)	0.0089 (19)
C38	0.114 (3)	0.102 (3)	0.103 (2)	0.046 (2)	0.071 (2)	0.024 (2)
C39	0.123 (3)	0.076 (2)	0.0821 (19)	0.047 (2)	0.053 (2)	0.0120 (16)
C40	0.090 (2)	0.0636 (18)	0.0726 (17)	0.0267 (15)	0.0361 (16)	0.0086 (13)

### Geometric parameters (Å, º)

**Table:**

O1—C12	1.229 (3)	C19—C20	1.379 (4)
O2—C32	1.228 (3)	C19—H19	0.9300
N1—C10	1.315 (3)	C20—H20	0.9300
N1—C1	1.369 (3)	C21—C26	1.409 (4)
N2—C30	1.317 (3)	C21—C22	1.413 (4)
N2—C21	1.365 (3)	C22—C23	1.349 (4)
C1—C6	1.408 (3)	C22—H22	0.9300
C1—C2	1.413 (3)	C23—C24	1.390 (5)
C2—C3	1.356 (4)	C23—H23	0.9300
C2—H2	0.9300	C24—C25	1.366 (4)
C3—C4	1.398 (4)	C24—H24	0.9300
C3—H3	0.9300	C25—C26	1.417 (3)
C4—C5	1.360 (4)	C25—H25	0.9300
C4—H4	0.9300	C26—C28	1.423 (3)
C5—C6	1.412 (3)	C27—C28	1.510 (4)
C5—H5	0.9300	C27—H27A	0.9600
C6—C8	1.426 (3)	C27—H27B	0.9600
C7—C8	1.510 (3)	C27—H27C	0.9600
C7—H7A	0.9600	C28—C29	1.371 (3)
C7—H7B	0.9600	C29—C30	1.426 (4)
C7—H7C	0.9600	C29—C32	1.512 (3)
C8—C9	1.374 (3)	C30—C31	1.506 (3)
C9—C10	1.426 (3)	C31—H31A	0.9600
C9—C12	1.508 (3)	C31—H31B	0.9600
C10—C11	1.507 (3)	C31—H31C	0.9600
C11—H11A	0.9600	C32—C33	1.451 (4)
C11—H11B	0.9600	C33—C34	1.318 (3)
C11—H11C	0.9600	C33—H33	0.9300
C12—C13	1.461 (4)	C34—C35	1.476 (4)
C13—C14	1.329 (3)	C34—H34	0.9300
C13—H13	0.9300	C35—C36	1.384 (4)
C14—C15	1.464 (3)	C35—C40	1.390 (3)
C14—H14	0.9300	C36—C37	1.380 (4)
C15—C16	1.385 (3)	C36—H36	0.9300
C15—C20	1.390 (3)	C37—C38	1.381 (4)
C16—C17	1.384 (4)	C37—H37	0.9300
C16—H16	0.9300	C38—C39	1.373 (4)
C17—C18	1.374 (4)	C38—H38	0.9300
C17—H17	0.9300	C39—C40	1.369 (4)
C18—C19	1.362 (4)	C39—H39	0.9300
C18—H18	0.9300	C40—H40	0.9300
			
C10—N1—C1	118.1 (2)	C15—C20—H20	119.8
C30—N2—C21	118.5 (2)	N2—C21—C26	122.9 (2)
N1—C1—C6	123.2 (2)	N2—C21—C22	117.6 (3)
N1—C1—C2	117.6 (2)	C26—C21—C22	119.5 (2)
C6—C1—C2	119.2 (2)	C23—C22—C21	120.9 (3)
C3—C2—C1	120.6 (3)	C23—C22—H22	119.5
C3—C2—H2	119.7	C21—C22—H22	119.5
C1—C2—H2	119.7	C22—C23—C24	120.4 (3)
C2—C3—C4	120.3 (3)	C22—C23—H23	119.8
C2—C3—H3	119.8	C24—C23—H23	119.8
C4—C3—H3	119.8	C25—C24—C23	120.5 (3)
C5—C4—C3	120.4 (3)	C25—C24—H24	119.8
C5—C4—H4	119.8	C23—C24—H24	119.8
C3—C4—H4	119.8	C24—C25—C26	121.0 (3)
C4—C5—C6	120.9 (3)	C24—C25—H25	119.5
C4—C5—H5	119.6	C26—C25—H25	119.5
C6—C5—H5	119.6	C21—C26—C25	117.8 (3)
C1—C6—C5	118.5 (2)	C21—C26—C28	117.7 (2)
C1—C6—C8	118.0 (2)	C25—C26—C28	124.5 (3)
C5—C6—C8	123.6 (2)	C28—C27—H27A	109.5
C8—C7—H7A	109.5	C28—C27—H27B	109.5
C8—C7—H7B	109.5	H27A—C27—H27B	109.5
H7A—C7—H7B	109.5	C28—C27—H27C	109.5
C8—C7—H7C	109.5	H27A—C27—H27C	109.5
H7A—C7—H7C	109.5	H27B—C27—H27C	109.5
H7B—C7—H7C	109.5	C29—C28—C26	118.7 (2)
C9—C8—C6	117.9 (2)	C29—C28—C27	121.8 (2)
C9—C8—C7	122.2 (2)	C26—C28—C27	119.6 (2)
C6—C8—C7	119.9 (2)	C28—C29—C30	119.7 (2)
C8—C9—C10	120.1 (2)	C28—C29—C32	121.7 (2)
C8—C9—C12	121.5 (2)	C30—C29—C32	118.4 (2)
C10—C9—C12	118.3 (2)	N2—C30—C29	122.5 (2)
N1—C10—C9	122.7 (2)	N2—C30—C31	116.8 (2)
N1—C10—C11	116.5 (2)	C29—C30—C31	120.6 (2)
C9—C10—C11	120.8 (2)	C30—C31—H31A	109.5
C10—C11—H11A	109.5	C30—C31—H31B	109.5
C10—C11—H11B	109.5	H31A—C31—H31B	109.5
H11A—C11—H11B	109.5	C30—C31—H31C	109.5
C10—C11—H11C	109.5	H31A—C31—H31C	109.5
H11A—C11—H11C	109.5	H31B—C31—H31C	109.5
H11B—C11—H11C	109.5	O2—C32—C33	120.3 (3)
O1—C12—C13	120.8 (3)	O2—C32—C29	118.8 (3)
O1—C12—C9	119.0 (3)	C33—C32—C29	120.9 (2)
C13—C12—C9	120.2 (2)	C34—C33—C32	124.5 (3)
C14—C13—C12	124.0 (2)	C34—C33—H33	117.7
C14—C13—H13	118.0	C32—C33—H33	117.7
C12—C13—H13	118.0	C33—C34—C35	126.4 (2)
C13—C14—C15	127.4 (2)	C33—C34—H34	116.8
C13—C14—H14	116.3	C35—C34—H34	116.8
C15—C14—H14	116.3	C36—C35—C40	118.2 (3)
C16—C15—C20	117.7 (3)	C36—C35—C34	118.7 (2)
C16—C15—C14	119.4 (2)	C40—C35—C34	123.0 (3)
C20—C15—C14	122.8 (3)	C37—C36—C35	121.0 (3)
C17—C16—C15	121.3 (3)	C37—C36—H36	119.5
C17—C16—H16	119.4	C35—C36—H36	119.5
C15—C16—H16	119.4	C36—C37—C38	119.6 (3)
C18—C17—C16	120.0 (3)	C36—C37—H37	120.2
C18—C17—H17	120.0	C38—C37—H37	120.2
C16—C17—H17	120.0	C39—C38—C37	119.9 (3)
C19—C18—C17	119.3 (3)	C39—C38—H38	120.1
C19—C18—H18	120.4	C37—C38—H38	120.1
C17—C18—H18	120.4	C40—C39—C38	120.4 (3)
C18—C19—C20	121.2 (3)	C40—C39—H39	119.8
C18—C19—H19	119.4	C38—C39—H39	119.8
C20—C19—H19	119.4	C39—C40—C35	120.8 (3)
C19—C20—C15	120.5 (3)	C39—C40—H40	119.6
C19—C20—H20	119.8	C35—C40—H40	119.6
			
C10—N1—C1—C6	0.1 (3)	C30—N2—C21—C26	−1.0 (4)
C10—N1—C1—C2	180.0 (2)	C30—N2—C21—C22	178.6 (2)
N1—C1—C2—C3	−179.9 (2)	N2—C21—C22—C23	−179.1 (2)
C6—C1—C2—C3	−0.1 (4)	C26—C21—C22—C23	0.6 (4)
C1—C2—C3—C4	1.0 (4)	C21—C22—C23—C24	−0.3 (5)
C2—C3—C4—C5	−1.2 (4)	C22—C23—C24—C25	0.1 (5)
C3—C4—C5—C6	0.4 (4)	C23—C24—C25—C26	−0.2 (4)
N1—C1—C6—C5	179.2 (2)	N2—C21—C26—C25	179.0 (2)
C2—C1—C6—C5	−0.6 (3)	C22—C21—C26—C25	−0.6 (4)
N1—C1—C6—C8	−1.1 (3)	N2—C21—C26—C28	0.1 (4)
C2—C1—C6—C8	179.1 (2)	C22—C21—C26—C28	−179.5 (2)
C4—C5—C6—C1	0.5 (4)	C24—C25—C26—C21	0.5 (4)
C4—C5—C6—C8	−179.2 (2)	C24—C25—C26—C28	179.2 (2)
C1—C6—C8—C9	1.2 (3)	C21—C26—C28—C29	0.9 (3)
C5—C6—C8—C9	−179.1 (2)	C25—C26—C28—C29	−177.9 (2)
C1—C6—C8—C7	−179.5 (2)	C21—C26—C28—C27	−179.4 (2)
C5—C6—C8—C7	0.1 (4)	C25—C26—C28—C27	1.8 (4)
C6—C8—C9—C10	−0.5 (3)	C26—C28—C29—C30	−1.0 (3)
C7—C8—C9—C10	−179.7 (2)	C27—C28—C29—C30	179.3 (2)
C6—C8—C9—C12	175.9 (2)	C26—C28—C29—C32	173.4 (2)
C7—C8—C9—C12	−3.3 (4)	C27—C28—C29—C32	−6.3 (4)
C1—N1—C10—C9	0.7 (4)	C21—N2—C30—C29	0.9 (4)
C1—N1—C10—C11	−179.6 (2)	C21—N2—C30—C31	179.8 (2)
C8—C9—C10—N1	−0.5 (4)	C28—C29—C30—N2	0.1 (4)
C12—C9—C10—N1	−177.0 (2)	C32—C29—C30—N2	−174.5 (2)
C8—C9—C10—C11	179.8 (2)	C28—C29—C30—C31	−178.8 (2)
C12—C9—C10—C11	3.4 (4)	C32—C29—C30—C31	6.6 (4)
C8—C9—C12—O1	−103.9 (3)	C28—C29—C32—O2	−97.8 (3)
C10—C9—C12—O1	72.5 (3)	C30—C29—C32—O2	76.7 (3)
C8—C9—C12—C13	75.3 (3)	C28—C29—C32—C33	82.5 (3)
C10—C9—C12—C13	−108.3 (3)	C30—C29—C32—C33	−103.1 (3)
O1—C12—C13—C14	−171.9 (3)	O2—C32—C33—C34	−169.0 (3)
C9—C12—C13—C14	8.9 (4)	C29—C32—C33—C34	10.8 (4)
C12—C13—C14—C15	−175.9 (2)	C32—C33—C34—C35	178.7 (2)
C13—C14—C15—C16	−179.9 (2)	C33—C34—C35—C36	176.9 (2)
C13—C14—C15—C20	3.5 (4)	C33—C34—C35—C40	−3.9 (4)
C20—C15—C16—C17	0.7 (4)	C40—C35—C36—C37	2.2 (4)
C14—C15—C16—C17	−176.0 (2)	C34—C35—C36—C37	−178.6 (2)
C15—C16—C17—C18	−0.6 (4)	C35—C36—C37—C38	−2.1 (5)
C16—C17—C18—C19	0.3 (4)	C36—C37—C38—C39	0.7 (5)
C17—C18—C19—C20	−0.1 (4)	C37—C38—C39—C40	0.4 (5)
C18—C19—C20—C15	0.2 (4)	C38—C39—C40—C35	−0.3 (4)
C16—C15—C20—C19	−0.5 (4)	C36—C35—C40—C39	−1.0 (4)
C14—C15—C20—C19	176.2 (2)	C34—C35—C40—C39	179.8 (2)

### Hydrogen-bond geometry (Å, º)

Cg1 and Cg2 are the centroids of the C1–C6 and C15–C20 rings, respectively.

**Table:**

*D*—H···*A*	*D*—H	H···*A*	*D*···*A*	*D*—H···*A*
C14—H14···N2	0.93	2.59	3.463 (3)	156
C7—H7*C*···*Cg*1^i^	0.96	2.86	3.662 (3)	142
C39—H39···*Cg*2^ii^	0.93	2.88	3.679 (3)	145

Symmetry codes: (i) −*x*, −*y*+1, −*z*+1; (ii) −*x*+1, −*y*+2, −*z*+2.
